# Patients with unexplained mismatch repair deficiency are interested in updated genetic testing

**DOI:** 10.1186/s13053-020-00150-1

**Published:** 2020-09-21

**Authors:** Jessica Omark, Eduardo Vilar, Y Nancy You, Leslie Dunnington, Sarah Noblin, Blair Stevens, Maureen Mork

**Affiliations:** 1 University of Texas MD Anderson Cancer Center UTHealth Graduate School of Biomedical Sciences, Houston, TX USA; 2 Department of Pediatrics, University of Michigan Health System Michigan Medicine, Ann Arbor, MI USA; 3 Department of Clinical Cancer Prevention, University of Texas MD Anderson Cancer Center, Houston, TX USA; 4 Department of Surgical Oncology, University of Texas MD Anderson Cancer Center, Houston, TX USA; 5 Department of Pediatrics, University of Texas McGovern Medical School, Houston, TX USA; 6 Department of Obstetrics, Gynecology, and Reproductive Sciences, University of Texas McGovern Medical School, Houston, TX USA; 7 Natera, San Carlos, CA USA; 8 Department of Clinical Cancer Genetics, University of Texas MD Anderson Cancer Center, Houston, TX USA

**Keywords:** Cancer, Genetic counseling, Genetic testing, Lynch syndrome, Mismatch repair deficiency, Oncology, Psychosocial impacts of genetic testing, Unexplained mismatch repair deficiency, Updated genetic testing

## Abstract

**Background:**

Individuals who have colorectal or endometrial cancers displaying loss of immunohistochemical staining of one or more mismatch repair proteins without an identifiable causative germline pathogenic variant have unexplained mismatch repair deficiency (UMMRD). Comprehensive germline genetic testing for Lynch syndrome (LS) includes sequencing and deletion/duplication analysis of *MLH1, MSH2, MSH6,* and *PMS2*, deletion analysis of *EPCAM*, and *MSH2* inversion analysis. Updated genetic testing to include elements of comprehensive LS testing not previously completed could further clarify LS status in individuals with UMMRD, allowing for tailored screening guidelines for affected individuals and their family members. However, patient understanding of the potential impact of updated genetic testing for LS is unclear. This study aimed to evaluate the interest in and perceived impact of updated genetic testing among individuals with UMMRD at a tertiary academic center.

**Methods:**

A survey evaluating interest in and perceived impact of updated genetic testing was mailed to 98 potential participants. Electronic health record review was completed for all individuals meeting eligibility criteria. Thirty-one individuals responded to the survey.

**Results:**

Results indicate this population is highly interested in updated genetic testing with the perceived impact being primarily for family members to have appropriate genetic testing and screening. Electronic health record review indicates that clinicians have an evolving understanding of causes of UMMRD, representing a potential change in assessment of cancer risk.

**Conclusions:**

Updated risk assessment and genetic counseling with a discussion of the benefits and limitations of germline and somatic genetic testing, is essential as the understanding of UMMRD and genetic testing recommendations for this population evolve.

## Background

Lynch syndrome (LS) is a hereditary cancer syndrome affecting as many as 1 in 279 individuals [1] characterized by an increased risk to develop colorectal cancer (CRC) and endometrial cancer (EC), as well as ovarian, stomach, small intestine, pancreatic, urinary tract, and brain cancers and sebaceous neoplasms [2]. LS is caused by a heterozygous germline pathogenic variant (PV) in one of four genes involved in the DNA mismatch repair (MMR) system: *MLH1, MSH2, MSH6,* and *PMS2.* Deletions of the *EPCAM* gene cause hypermethylation of the *MSH2* promoter region and are also associated with LS [3]. Inversion of *MSH2* exons 1–7 is another known cause of LS that cannot be detected by traditional sequencing or deletion/duplication analysis [4].

CRC and EC in individuals with LS typically display high levels of microsatellite instability (MSI) and/or show loss of immunohistochemical (IHC) staining of one or more MMR proteins, most frequently corresponding with the germline PV. If a CRC or EC exhibits loss of function of the DNA MMR system, genetic testing (GT) is recommended to determine if there is a germline PV causing LS [5, 6].

In approximately 2–4% of patients with CRC, IHC staining indicates MMR protein loss, but GT does not detect a germline PV [7, 8]. This situation is known as unexplained mismatch repair deficiency (UMMRD) [7, 9]. Recent studies have shown that acquired biallelic somatic MMR inactivation in the tumor explains the loss of protein staining in 45–69% of individuals with UMMRD [7, 10] and is not associated with a genetic predisposition to cancer. Guidelines from the National Comprehensive Cancer Network (NCCN) indicate that paired tumor and germline GT may be recommended to evaluate for biallelic somatic MMR inactivation depending on IHC findings [5], and paired tumor and germline GT has become clinically available [11].

However, the etiology continues to be unknown for the remaining 31–55% of individuals with UMMRD. Some of these cases may be caused by a germline PV that was not detected by the original GT. The suspicion for a previously undetected germline PV is especially high for patients meeting Amsterdam criteria for the identification of individuals likely to have LS [12]. NCCN guidelines indicate that patients with UMMRD and their family members should be managed based on the family history of cancer [5]. There are separate screening guidelines in place for individuals with a molecular diagnosis of LS [5, 6]. A survey of screening practices of family members of individuals with UMMRD indicates that a minority of family members are currently following LS surveillance [9]. Therefore, identification of a germline PV causing LS is likely to change current surveillance practices of family members.

Traditional GT for LS was based on the pattern of protein loss on IHC staining (i.e., *MSH2* analysis for absence of MSH2/MSH6 protein staining). This strategy can fail to detect a germline PV if the causative variant cannot be detected by sequencing and deletion/duplication analysis of MMR genes, such as the known *MSH2* inversion, or if IHC analysis is false-normal for a specific MMR protein, indicating that the staining is intact while the tumor is deficient for an additional MMR protein [13]. Additionally, a small portion of LS-related tumors will not show evidence of MMR deficiency [13]. Some cases of UMMRD may be attributed to a PV in a gene associated with a separate hereditary cancer syndrome such as *MUTYH, POLD1,* or *POLE* [14, 15].

For these reasons, as well as decreased cost of testing multiple genes via next-generation sequencing (NGS) panel GT, patients suspected to have LS based on tumor GT are now frequently offered sequencing and deletion/duplication analysis of all LS-related genes, *MSH2* inversion analysis, and deletion/duplication analysis of *EPCAM*. Therefore, updated comprehensive GT for LS or additional hereditary CRC predisposition syndromes may be appropriate for individuals who previously had incomplete GT.

Previous studies evaluating the implications of updated GT for other genetic syndromes using multi-gene panel testing have cited the increased chance for identification of a genetic diagnosis [16], as well as the increased risk for the identification of a variant of uncertain significance (VUS) [17]. However, there is limited information available regarding the patient perspective surrounding consideration of updated GT.

Cancer genetics clinicians are aware of the importance of comprehensive evaluation for germline PVs in order to provide appropriate risk assessment and screening recommendations. However, it is also essential that affected individuals understand the potential impacts of identifying a germline PV. If patients do not understand the potential impact of updated GT for themselves or their family members, they may fail to receive updated GT or to communicate these results to relatives. Therefore, this study aims to evaluate the interest in and perceived impact of updated germline GT for LS among individuals with UMMRD.

## Methods

The study population consisted of patients from the University of Texas MD Anderson Cancer Center (UTMDACC) with a personal history of CRC or EC and UMMRD detected by loss of IHC staining without presence of a PV upon incomplete clinical germline GT for LS. This included patients with one or more VUSs. Complete clinical germline GT was defined as sequencing and deletion/duplication analysis of *MLH1, MSH2, MSH6,* and *PMS2,* deletion/duplication analysis of *EPCAM*, and *MSH2* inversion testing. MSI analysis was not completed for all individuals, and therefore MSI results were not utilized as an eligibility criterion.

All study participants were English-speaking and 18 years or older. Individuals meeting the inclusion criteria were identified by querying the UTMDACC genetic counseling database. Exclusion criteria included individuals with tumors showing loss of IHC staining for MLH1 with *BRAF* V600E mutation or *MLH1* promoter hypermethylation, as these events are consistent with sporadic tumors. Tumor GT to evaluate for biallelic somatic MMR inactivation was not completed at the time of cancer diagnosis, as it was not widely clinically available at the time. Eligibility criteria was confirmed by evaluating patient electronic health records (EHR). The study was approved by the UTMDACC Institutional Review Board (PA17–0473) and the University of Texas Health Science Center at Houston Institutional Review Board (HSC-MS-17-0831).

A survey developed by the authors (Additional file 1) containing questions about current screening behaviors, original GT considerations, interim family histories, and the perceived impact of updated GT results was utilized. Questions included multiple choice, Likert scale, rank-order, and open-ended response questions.

The survey and informed consent were mailed to each individual meeting eligibility criteria with information about the availability of updated germline GT as part of clinical care. The informed consent document provided consent for survey participation as well as review of EHR. Up to three attempts were made to contact the participants. Participants also had the option to complete the survey over the phone or when approached while attending scheduled clinic visits.

An EHR review was completed for each individual meeting eligibility criteria. The information obtained included basic demographic information, personal and family history of cancer, dates of genetic counseling visits, tumor pathology results, and GT results.

Deidentified survey responses and data collected from the EHR were entered in the online survey tool RedCap. Patient data were stored on a secure server hosted by UTMDACC. Descriptive statistics were used to characterize all variables of interest. Survey respondents were compared to non-respondents who met eligibility criteria for the study. Categorical demographic variables were compared using chi-square analysis and Fischer’s exact test. Independent-sample t-tests were conducted to compare continuous variables between survey respondents and non-respondents. Statistical tests were performed using STATA v.14 (College Station, TX). A *p* value of < 0.05 was considered statistically significant.

## Results

A total of 97 patients met the eligibility criteria, of whom 31 individuals responded to the survey (response rate of 32%). There were no statistically significant differences between respondents and non-respondents (Additional file 2) when comparing sex (*p* = 0.729), ethnicity (*p* = 0.105), cancer diagnosis (*p* = 0.240), cancer history (*p* = 0.980), GT results (*p* = 0.760), or family history (*p* = 0.913). There was no significant difference between the ages of respondents (mean = 60.2; SD = 11.60) and non-respondents (mean = 55.0; SD = 11.96); t (95) = 1.649, *p* = 0.103. There was also no difference between the years since last contact with a genetic counselor of respondents (mean = 5; SD = 2.91) and non-respondents (mean = 6; SD = 3.41); t (95) = 1.867, *p* = 0.0661).

Surveys were initially mailed to 98 individuals, one of whom was determined to be deceased based on response from a family member; this individual was not included in the group of non-respondents.

The demographic characteristics of the respondents are summarized in Table 1. Overall, the population was largely non-Hispanic white (81%), attained at least some college education (87%), and had an annual household income greater than $50,000 (68%). The average respondent age was 60.2 years (range 33–81 years), and the average number of children was 2 (range 0–5).

**Table 1:** Respondent Demographics

	N out of 31 (%)
**Ethnicity**^a^	
Non-Hispanic White	25 (81)
Hispanic	2 (6)
Asian	1 (3)
Other	3 (10)
**Education**^a^	
< High School	1 (3)
High School/GED	3 (10)
Associate/Bachelor	10 (32)
Postgraduate degree	17 (55)
**Religion**^a^	
Christian	29 (94)
Do not identify	1 (3)
Hinduism	1 (3)
**Annual Household Income**^a,b^	
< $50,000	5 (16)
$50,000-100,000	8 (26)
> $100,000	13 (42)
**Sex**^c^	
Female	19 (61)
Male	12 (39)
**Average Age**^**c**^	60.2 years (range of 33-81)

^a^Based on respondent self-identification

^b^Five respondents declined to respond

^c^Collected from the EHR

### Cancer history

Twenty-one (68%) respondents had a personal history of CRC, and 10 (32%) of EC. Nine (29%) respondents additionally had a personal history of another cancer including breast, prostate, skin, and stomach tumors. One of these nine respondents had a personal history of colorectal, skin, and stomach cancers and a sebaceous neoplasm.

Six (19%) respondents did not have MSI analysis results; the remaining individuals were divided between MSI-high (74%) and microsatellite-stable (6%). IHC results of CRC or EC were abnormal in all 31 respondents (Table 2). Twenty (65%) respondents had tumors displaying loss of more than one protein.

**Table 2:** IHC Results

	N out of 31 (%)
Absent MLH1 only	2
Absent MSH6 only	5
Absent PMS2 only	20
Absent MSH2 and MSH6	7
Absent MLH1 and PMS2	12
Absent MSH6 and PMS2	1
Indeterminate IHC	1

Original GT consisted of analysis of one gene for 13 (42%) and analysis of two genes for 9 (29%) respondents. The remaining respondents had analysis of more than two genes on original GT. Twenty-three (74%) respondents had uninformative negative results upon original GT, while 8 (26%) respondents had a VUS. The time from initial GT to the time of survey completion ranged from 1 year to 14 years, with a median of 6 years. The most common GT strategy at the performing institution during that timeframe was single-gene testing.

### Family history of cancer

Based on analysis of EHR review and survey responses, 4 (13%) respondents met Amsterdam criteria. Twenty-nine (94%) respondents had a family history of cancer at the time of original genetic counseling. Of these, 23 (79%) had a family history of at least one LS-related cancer. Of the two respondents without a family history of cancer, one had no family history of cancer and the other was adopted. In an evaluation of the reported interim family history of cancer, 10 (32%) respondents had a family history of cancer since original genetic counseling. Of these, 4 (40%) respondents had an interim family history of at least one LS-related cancer, while 6 (60%) respondents had a family member diagnosed with non-LS-related cancer since original genetic counseling.

### Perceived cause of cancer

**Fig. 1:**
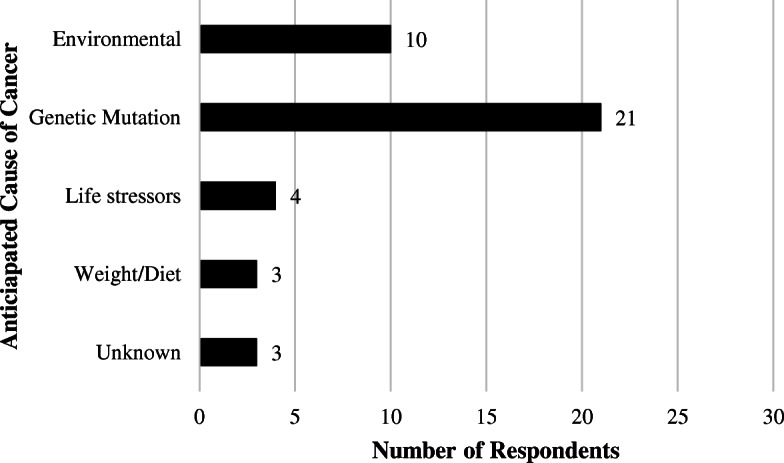
Perceived cause of colorectal cancer (CRC) or endometrial cancer (EC) among survey respondents. Respondents were able to select more than one perceived cause

Respondents were asked what they believed to be the cause of their cancer (Fig. 1—Respondent-Perceived Cause of CRC or EC). Eight (26%) respondents thought there was more than one cause for their cancer. Twenty-one (68%) respondents thought a germline PV was at least one reason for the development of cancer. Twenty-seven (87%) respondents thought it was important or extremely important to determine the cause of their cancer. In an open-ended question format, 15 (48%) individuals stated concern for family members as a reason for the importance of determining the cause of the original cancer. This was the single most frequently stated reason.

EHR review indicated 7 (23%) respondents were told they have a definitive diagnosis of LS based on IHC results. Nine (29%) respondents were told they likely have LS, while 14 (45%) respondents were told it is unclear whether they have LS. One respondent was told that LS was an unlikely explanation for the IHC results. Of the 9 respondents who were told they likely have LS, 8 (89%) perceived a germline PV be an underlying cause of their EC or CRC.

### Interest in original GT

**Fig. 2:**
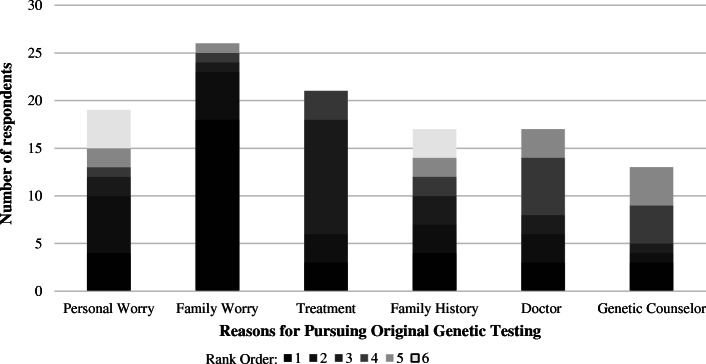
Factor in rank order 1 is the factor that most highly influenced the decision to originally undergo genetic testing. If respondents did not feel a listed factor influenced their original genetic testing decision, it was not ranked. Factors for undergoing testing include: concern for personal risk to develop cancer, concern for family member risk to develop cancer, optimizing cancer treatment, concern based on family history of cancer, doctor recommendation, and genetic counselor recommendation

Twenty-six respondents (84%) indicated the original decision to undergo GT was either not stressful or a little stressful. Eighteen (58%) respondents indicated their primary reason for pursuing GT was concern that other family members may develop cancer (Fig. 2—Factors Contributing to Decision to Undergo Original Genetic Testing). The most frequently ranked second answer was concern for an increased risk to develop another LS-related cancer. Association between concern for an increased risk to develop another cancer and current age (*p* = 0.79) was not statistically significant.

### Interest in updated GT

Twenty-four (77%) respondents indicated they were either interested or extremely interested in updated GT. In an open-ended question format, 11 individuals cited providing information to family members as a reason for interest in updated GT; this was the most frequently cited factor. Twenty-three (74%) respondents were at least somewhat worried that family members would develop cancer, and 13 (42%) respondents were very worried that family members would develop cancer.

**Fig. 3:**
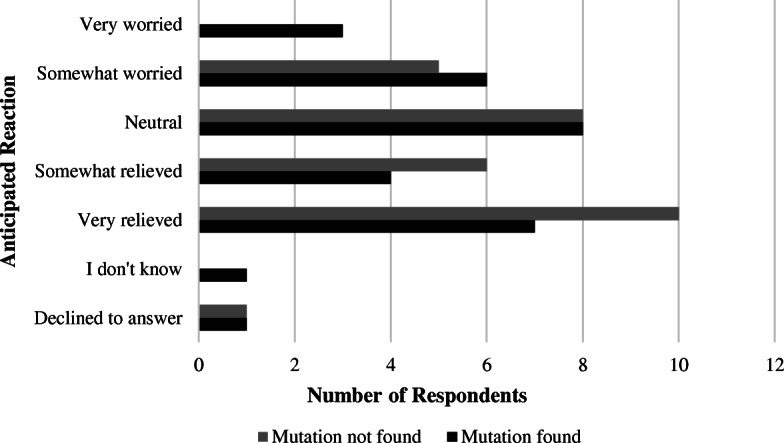
Anticipated reaction to results presuming that a pathogenic variant was identified or not identified on updated genetic testing

When asked about expected feelings toward results of updated GT, (Fig. 3-Participants’ Anticipated Reactions to Results of Updated Genetic Testing), 7 (23%) respondents indicated they would feel very relieved if GT identified a PV consistent with LS, while 10 (32%) respondents indicated they would feel very relieved if updated GT were negative. In comparison, 3 (10%) respondents indicated they would feel very worried if a PV was found on updated GT, whereas no respondents indicated they would feel very worried if no PV was found. Overall, 14 (45%) respondents indicated they would feel relatively less concerned or more relieved if a PV were not identified on updated GT. This was not a statistically significant difference (*p* = 0.21). The most frequently cited reason for relief if a PV were identified was the implementation of appropriate screening for family members.

When asked in open-ended question format about concerns regarding updated GT, 23 (74%) respondents did not have any concerns. Those respondents who did have concerns cited time requirements for GT, concerns about the impacts of the results, and concerns about insurance coverage and privacy.

### Cancer screening practices

Fifteen (48%) respondents indicated they undergo colonoscopies at least annually. Of the 9 female respondents with a history of CRC, 4 (44%) have had a total hysterectomy and bilateral salpingo-oophorectomy, 3 (33%) had a hysterectomy only, and 2 (22%) had their uterus and ovaries intact. The reasons for surgeries were not elicited. Of the 10 women who had EC, 80% had colonoscopies at least every 2–3 years.

When asked about perceived frequency of colonoscopies if a germline PV were identified on updated GT, 18 of 31 (58%) respondents indicated that they would have colonoscopies at the same frequency, while 11 (35%) respondents thought the frequency of colonoscopies would increase.

## Discussion

Our study aimed to evaluate the interest in and perceived impact of updated GT among patients with UMMRD. The results emphasize that the primary reason for interest in updated GT is concern for family members to develop cancer and desire for family members to have appropriate screening. Providing family members with accurate information was the most frequently stated reason for interest in updated GT and the most frequently stated reason for the importance to determine the cause of cancer. Respondents indicated they were concerned for family members to develop cancer at the time of original GT, and 74% of respondents remain at least somewhat worried about family members developing cancer. Previous studies evaluating the motivators for GT for LS have found that concern for family members is frequently a motivating factor [18, 19]. Therefore, these findings suggest that the reasons for interest in updated GT among this population are similar to those indicated in the literature for original GT for LS.

Respondents indicated they expected to feel relief for family members after updated GT, regardless of the result. Respondents who indicated they would feel relief if a germline PV was found on updated GT often cited that family members could have GT and high-risk surveillance if found to be positive for the familial PV. Concern for family members was not mentioned as a possible deterrent for updated GT. It may be that information gained from updated GT is viewed as being helpful for family members, regardless of the results.

Because respondents are focused primarily on impacts of GT for family members, it may be important for clinicians to emphasize the potential implications of updated GT for patients themselves. This population had an increased frequency of colonoscopies as compared to the general population recommendations. Frequent colonoscopies may be expected for individuals with a personal history of CRC. However, women with EC and no personal or family history of CRC who are receiving frequent colonoscopies are having more screening than would be recommended if updated GT and risk assessment were suggestive of a sporadic cancer rather than LS. Additionally, women with UMMRD who have a personal history of CRC may have been encouraged to consider hysterectomy/oophorectomy if thought to have LS. Overall, 77% of female respondents with a history of CRC had a risk-reducing surgery for endometrial and/or ovarian cancer. While the reasons for these risk-reducing surgeries were not elicited, the high surgical rate may indicate a perceived increased risk for gynecologic cancers. Women with LS are recommended to undergo risk-reducing surgeries, while women with UMMRD are not [5]. Therefore, the potential impact of updated GT on the management recommendations for women with UMMRD should be emphasized.

The results of this study also indicate a need for updated genetic counseling among individuals with UMMRD. There was wide variation in respondents’ anticipated feelings toward results of updated GT. If updated GT were negative, many respondents expected to feel relief because family members could have less frequent screening, while other respondents expected to feel worried because they would still not know the cause of the cancer. An updated review of medical and family histories and review of additional germline and somatic GT options may allow for identification of the underlying cause of cancer in individuals with UMMRD.

Implications of GT results are especially nuanced for individuals who had a VUS upon original GT and/or for those who meet Amsterdam criteria. Therefore, updated genetic counseling may be especially important for these subgroups of respondents. For individuals meeting Amsterdam criteria who had a VUS on original GT, the VUS could potentially represent a PV. Patients with a VUS in a gene associated with LS have been shown to have concerns regarding appropriate cancer screening for family members [20]. Updated genetic counseling should include a reevaluation of these variants for potential updates in classification. The respondents who did not meet Amsterdam criteria may have other explanations for MMR defects in their tumors.

Perhaps the most important reason for updated genetic counseling in this population is to provide updates about clinician understanding of potential causes of MMR deficiency. Until approximately 2014, the primary cause of tumor defects in the MMR pathway (other than *MLH1* promoter hypermethylation or *BRAF* V600E mutation) was thought to be LS, and patients were often counseled that they likely had LS even in the absence of a germline PV. While 45–69% of patients with UMMRD are expected to have biallelic somatic MMR inactivation causing MMR deficiency, [7, 10] 68% percent of respondents perceived at least one cause of their cancer to be a germline PV. It is unlikely that all the respondents who perceive that a germline PV caused their cancer truly have LS.

With a range of between 1 and 14 years passing between time of original GT and time of survey response, improvements in GT technology and increased understanding of possible etiologies of UMMRD have resulted in NGS panel-based germline GT for hereditary cancer syndromes and guidelines indicating consideration of paired somatic/germline GT [5], representing a distinct change in GT strategy. This may require re-contacting patients with UMMRD, including those who previously had comprehensive germline GT. Previous studies have shown that patients believe it is important to know about updates in available GT for other cancer types, but the most effective method for notifying patients about updated GT remains unclear [21]. Comparison of demographics and previous test results between respondents and non-respondents did not identify any significant differences, indicating that most individuals with UMMRD may be open to re-contact, and that methods other than a mailed letter may be more effective in eliciting interest. Given the rapid changes in germline and tumor GT recommendations for individuals with UMMRD, updated genetic counseling with or without updated GT can provide patients with current information regarding the evolving understanding of the clinical significance of MMR deficiency and the most appropriate screening recommendations.

### Study limitations

Our population was overall highly educated, with a non-Hispanic white background and an average age of 60.2-years-old, largely reflecting the average patient population at this tertiary cancer center. It is not clear if the results of the study can be extrapolated to individuals of other backgrounds or younger individuals who may have different perceptions of their personal cancer risks. Our population was subject to selection bias, as individuals interested in updated GT may be more likely to respond to a survey on this subject. Our study is limited by a low response rate and low statistical power. It is possible that the low response rate is influenced by the time (median of 6 years) between original GT and the mailing of the surveys. Although UTMDACC is a large tertiary care center with an extensive patient database, only 97 individuals met eligibility criteria, reflecting the specificity of the criteria.

## Conclusions

Genetic counselors should provide an updated cancer risk assessment, discussion of potential causes of MMR deficiency with consideration of additional clinically appropriate GT, and updated screening recommendations based on current clinical understanding of UMMRD. This study provides genetic counselors with additional information about the psychosocial concerns of this patient population, especially regarding concern for family members as a primary motivating factor for updated GT. Genetic counselors should validate these psychosocial concerns and elicit individual beliefs regarding updated GT when providing updated genetic counseling.

### Research recommendations

Our population was primarily highly educated and non-Hispanic white; further research is necessary to elucidate if similar concerns are prevalent across other socioeconomic backgrounds. A cross-institutional study of individuals with UMMRD may provide adequate statistical power to establish factors that contribute to interest or lack of interest in updated GT and a further investigation of subgroups of interest, including women with risk-reducing surgeries, those who fulfill Amsterdam criteria, and/or those with a VUS upon original GT. A survey of patient attitudes toward somatic MMR testing and consideration of the cost-effectiveness of such testing is critical as paired tumor/germline testing is integrated into the GT landscape.

## Supplementary information

**Additional file 1.**

**Additional file 2.**

## Data Availability

The datasets used and/or analyzed during this study are available from the corresponding author on reasonable request.

## Abbreviations

CRC: Colorectal cancer
EC: Endometrial cancer
EHR: Electronic health record
GT: Genetic testing
IHC: Immunohistochemical
LS: Lynch syndrome
MMR: Mismatch repair
MSI: Microsatellite instability
NCCN: National Comprehensive Cancer Network
NGS: Next-generation sequencing
PV: Pathogenic variant
UMMRD: Unexplained mismatch repair deficiency
UTMDACC: University of Texas MD Anderson Cancer Center
VUS: Variant of uncertain significance
