# Supplementary material for: Patients with unexplained mismatch repair deficiency are interested in updated genetic testing
Source: Hered Cancer Clin Pract. 2020 Sep 21;18:19. doi: 10.1186/s13053-020-00150-1 (PMC7507605; doi:10.1186/s13053-020-00150-1)
Supplement: Supplementary file 1 — Additional file 1. [file 13053_2020_150_MOESM1_ESM.docx]

**Title: Patient Perceptions of Germline Mutation Findings in People with Unexplained Mismatch Repair Deficiency**

**Survey**

You are receiving this survey because you were evaluated for Lynch syndrome based on previous testing on your colorectal or endometrial tumor. Lynch syndrome is a genetic condition that leads to an increased risk for colorectal, uterine (endometrial), and other cancers. You had genetic testing for Lynch syndrome, but results came back negative. This means we did not find a genetic change that explained why you developed cancer. You have received a letter explaining that there is now updated genetic testing available to you.

The following survey aims to evaluate your views and opinions about additional testing to determine if you have Lynch syndrome. If you decide to take part in the study, your total time commitment is estimated to be 15 minutes. You can refuse to answer any questions asked or written on any forms. Participation in this study is voluntary. A decision not to take part in this study will not change the services you receive through MD Anderson Cancer Center.

**Please answer the following questions as completely as possible:**

**Demographics**

1. With which ethnicity do you identify?

- Black
- Caucasian
- Hispanic
- Asian
- Other (please specify):

1. What is the highest level of education that you have completed?

- Did not finish high school
- High school/Equivalent
- Associate’s degree (2 years of college)
- Bachelor’s degree (4 years of college)
- Master’s degree/PhD/Professional degree (MD, JD)
- Trade school
- Other (please specify):

1. How many biological children do you have?
2. With which religious belief system do you identify?

- Christian
- Muslim
- Judaism
- I do not identify with a religion
- Other: (please specify)

5. Do you have health insurance from a private company (such as Cigna, BlueCross BlueShield) or from a public source (such as Medicare or Medicaid)? Please circle one option below.

**PUBLIC INSURANCE PRIVATE INSURANCE I DON’T HAVE INSURANCE**

6. What is your total annual **household** income before taxes?

- Less than $10,000 per year
- $10,000-$24,999 per year
- $25,000-$49,999 per year
- $50,000-$74,999 per year
- $75,000-$99,999 per year
- Greater than $100,000 per year

**Family History**

7. Since the time that you originally had genetic counseling and genetic testing, have any biological family members (parents, siblings, children, aunts/uncles, grandparents, cousins) been diagnosed with cancer? If so, please list them below including the relationship to you, the type of cancer, and the age of the family member when he/she was diagnosed.

**Family Member Type of Cancer Age at Diagnosis**

| *Example: Brother* | *Colon Cancer* | *56* |
| --- | --- | --- |
|  |  |  |
|  |  |  |
|  |  |  |
|  |  |  |

If you do not have any family members who have been diagnosed with cancer since the time of your original genetic counseling, please check here: _______

**Previous Genetic Testing**

1. What do you think caused your cancer? Please check all that apply.

- Environmental exposures
- Genetic mutation
- Life stressors
- Smoking
- Diet/weight
- Other (please explain):

1. What was your original reason for pursuing genetic testing for Lynch syndrome? Please rank the following reasons, with “1” being the most important reason to you. If any of the reasons do not apply, please write “N/A.”

____ I was worried about getting cancer again

____ I was worried about my family members getting cancer

___ To determine the best treatment or screening regimen

____ My family history of cancer

____ My doctor told me to

____ My genetic counselor told me to

____ Other (please explain):

1. How stressful or worrisome was it for you to decide to have genetic testing for Lynch syndrome originally?

1 2 3 4 5

Not stressful A little stressful Neutral Stressful Extremely Stressful

Please explain:

1. How important to you is it to find out what caused your cancer?

1 2 3 4 5

Not important A little important Neutral Important Extremely Important

Please explain:

1. We have not currently found a genetic change that explains why you developed cancer. Based on your negative test results, do you think your family members would currently be recommended to pursue genetic testing?

**YES NO**

12a. If so, for which living family members do you think genetic testing would currently be recommended?

- Parents
- Siblings
- Children
- Aunts/Uncles
- Nieces/Nephews
- Cousins
- Grandparents
- Other:

1. Have any of your family members already undergone genetic testing for a hereditary cancer syndrome?

**YES NO I DON’T KNOW**

13a. If so, what is this person’s relationship to you, and what were the results of the test?

1. Based on your history of cancer, do you think any of your family members currently wish to pursue genetic testing?

**YES NO I DON’T KNOW**

Comments:

1. How concerned are you that your family members may also get cancer?

1 2 3 4 5

Not worried A little worried Somewhat worried Moderately Worried Very Worried

Comments:

1. How frequently do you receive colonoscopies?

- Multiple times a year
- Once a year
- Every 2-3 years
- Every 4-5 years
- Every 6-10 years
- Never

1. Do you have any other regular screening to check for cancer?

- Mammogram/breast exam
- Prostate cancer blood test
- Ovarian cancer blood test
- Upper endoscopy
- Other:

**Updated Genetic Testing**

1. Updated genetic testing is available to you. Are you interested in pursuing further genetic testing that could identify a cause for your cancer?

1 2 3 4 5

Not interested A little interested Neutral Interested Extremely Interested

If interested, why? If not interested, why not?

1. How would you feel **if a genetic mutation were found** in the updated testing?

1 2 3 4 5

Very worried Somewhat worried Neutral Somewhat relieved Very relieved

Comments:

1. If you were found to have a mutation that explained your cancer, with whom would you share this information? Check all that apply

- Spouse/partner
- Family: parents, siblings, children, etc.
- Friends
- Healthcare provider
- A spiritual leader
- A support group
- Other (please list):

1. How do you think you would feel **if no genetic mutation were found** in the updated testing?

1 2 3 4 5

Very worried Somewhat worried Neutral Somewhat relieved Very relieved

Comments:

1. **If a mutation was found** that predisposed you to develop cancer, do you think your family members would be recommended to pursue genetic testing for a predisposition to develop cancer?

**YES NO**

22a. Based on these test results, to which family members do you think genetic testing for a predisposition to develop cancer would be recommended?

- Parents
- Siblings
- Children
- Aunts/Uncles
- Nieces/Nephews
- Cousins
- Other:
- None of my relatives

1. **If no mutation was found** that predisposed you to develop cancer, do you think your family members would be recommended to pursue genetic testing for a predisposition to develop cancer?

**YES NO**

23a. Based on these test results, to which family members do you think genetic testing for a predisposition to develop cancer would be recommended?

- Parents
- Siblings
- Children
- Aunts/Uncles
- Nieces/Nephews
- Cousins
- Other:
- None of my relatives

1. How do you think your colonoscopy screening would be different if we found a mutation that caused your colorectal cancer?

- More frequent colonoscopies
- Same number of colonoscopies
- Less frequent colonoscopies

1. If a mutation were found that explained why you developed cancer, what other types of screening do you think would be recommended? Check all that apply.

- Skin exam
- Mammogram/breast exam
- Prostate cancer blood test
- Ovarian cancer blood test
- Upper endoscopy
- Other:

1. Do you have any concerns about pursuing further genetic testing?

**YES NO UNSURE**

1. If so, what are they?

Thank you for participating in this survey. Please place the completed survey and consent form in the prepaid envelope included with this package, and place it in the mail to be returned to MD Anderson. If you have any questions or concerns regarding this study, please contact Maureen Mork or Jessica Omark, members of the study team, at: 713-745-4013.
