# Supplementary material for: Patients with unexplained mismatch repair deficiency are interested in updated genetic testing
Source: Hered Cancer Clin Pract. 2020 Sep 21;18:19. doi: 10.1186/s13053-020-00150-1 (PMC7507605; doi:10.1186/s13053-020-00150-1)
Supplement: Supplementary file 2 — Additional file 2. [file 13053_2020_150_MOESM2_ESM.docx]

|  | **Respondents (N=31)** | **Non-respondents (N=66)** | **p-value** |  |  |  |
| --- | --- | --- | --- | --- | --- | --- |
| **Sex** |  |  | 0.729 |  |  |  |
| Female | 19 (61) | 38 (58) |  |  |  |  |
| Male | 12 (39) | 28 (42) |  |  |  |  |
| **Race/Ethnicity** |  |  | 0.105 |  |  |  |
| Non-Hispanic White | 25 (81) | 48 (73) |  |  |  |  |
| Hispanic | 2 (6) | 4 (6) |  |  |  |  |
| Black | 0 (0) | 4 (6) |  |  |  |  |
| Asian | 1 (3) | 9 (14) |  |  |  |  |
| Other | 3 (10) | 1 (1) |  |  |  |  |
| Avg age at survey (range) | 62 (33-81) | 56 (35-82) | 0.103 | Df = 95 | t= 1.649 | SD=11.943 |
| Avg years since last GC contact (range) | 5 (1-11) | 6 (1-16) | 0.0661 | Df = 95 | t= 1.867 | SD = 3.29 |
| **Cancer diagnosis** |  |  | 0.24 |  |  |  |
| CRC | 21 (68) | 52 (79)* |  |  |  |  |
| Endometrial | 10 (32) | 15 (23)* |  |  |  |  |
| **History of two or more cancers** |  |  | 0.98 |  |  |  |
| Yes | 9 (29) | 19 (29) |  |  |  |  |
| No | 22 (71) | 47 (71) |  |  |  |  |
| **Variant of uncertain significance** |  |  | 0.76 |  |  |  |
| Yes | 8 (26) | 18 (27) |  |  |  |  |
| No | 23 (74) | 48 (73) |  |  |  |  |
| **Family history meets AI/AII** |  |  | 0.913 |  |  |  |
| Yes | 4 (13) | 8 (12) |  |  |  |  |
| No | 27 (87) | 58 (88) |  |  |  |  |
| * one with both CRC and endometrial cancer | |  |  |  |  |  |
